# Functional and structural retinal alterations in Alzheimer’s disease: insights from photopic negative response (PhNR) and OCT-based analysis

**DOI:** 10.1007/s10633-026-10089-8

**Published:** 2026-03-04

**Authors:** Hidayet Sener, Furkan Ozer, Osman Ahmet Polat, Beyza Ogurlu, Hatice Kubra Sonmez, Murat Gultekin, Fatih Horozoglu

**Affiliations:** 1 Department of Ophthalmology, Erciyes University Medical Faculty, Kayseri, Turkey; 2 Nevsehir State Hospital, Nevsehir, Turkey; 3 Department of Neurology, Erciyes University Medical Faculty, Kayseri, Turkey

**Keywords:** Alzheimer’s disease, Photopic negative response, Electroretinography, Retinal ganglion cells, Optical coherence tomography, Retinal nerve fiber layer

## Abstract

**Background:**

We evaluated retinal function and structure in Alzheimer’s disease (AD) using photopic negative response (PhNR) from full-field electroretinography (ERG) and using optical coherence tomography (OCT)-based retinal layer measurements.

**Methods:**

Ninety eyes from 61 subjects (31 controls, 30 AD patients) were included. Full-field ERGs were recorded using chromatic red-on-blue stimulation (1.7 cd·s/m^2^ flashes over an 8 cd/m^2^ blue background) in accordance with the ISCEV extended PhNR protocol. The AD group included 16 females and 14 males with moderate cognitive impairment (mean MMSE 15.5 ± 3.9). ERG a-wave, b-wave, and PhNR amplitudes were recorded. Macular and peripapillary retinal nerve fiber layer (RNFL) thicknesses were measured by OCT.

**Results:**

A-wave (AD: − 13.4 ± 11.9 µV, control: − 32.5 ± 13.7 µV, *p* < 0.001), b-wave (AD: 59.1 ± 28 µV, control: 156 ± 48.8 µV, *p* < 0.001), PhNR (AD: − 21.4 ± 14.9 µV, control: − 51 ± 15.4 µV, *p* < 0.001) amplitudes were lower in AD. Thicknesses of the central INL (AD: 25 ± 6.6 µm, control: 25.2 ± 8.2 µm, *p* = 0.04), inferior OPL (AD: 32.7 ± 6.2 µm, control: 36.9 ± 8.9 µm, *p* = 0.01), and temporal OPL (AD: 29.3 ± 5.2 µm, control: 34.8 ± 7.0 µm, *p* = 0.001) were reduced in AD, while other retinal layers showed no significant differences between groups (*p* > 0.05). The b-wave amplitude showed the highest discriminative power with an AUC of 0.956, followed by the PhNR amplitude (AUC = 0.930) and the a-wave amplitude (AUC = 0.904).

**Conclusion:**

AD was associated with a generalized reduction in full-field ERG amplitudes, accompanied by selective retinal layer changes on OCT. Among electrophysiological measures, b-wave amplitude demonstrated the highest ability to distinguish AD patients from controls.

**Supplementary Information:**

The online version contains supplementary material available at 10.1007/s10633-026-10089-8.

## Introduction

Alzheimer’s disease (AD) is the most common dementia subtype, characterized by progressive cognitive impairment and extensive neurodegeneration [1]. Accumulating evidence indicates retinal changes that parallel brain pathology in AD, suggesting the retina as a potential biomarker site for noninvasive detection of neurodegeneration [2, 3]. Due to embryological and anatomical similarities between retina and brain, retinal biomarkers might provide early diagnostic insights for AD [4].

Electroretinography (ERG) is widely used to assess retinal function; particularly, the photopic negative response (PhNR), generated mainly by retinal ganglion cells and their axons, is sensitive for detecting inner retinal dysfunction [5]. Although previous studies demonstrated decreased PhNR amplitudes in other neurodegenerative disorders, including Parkinson’s disease, data regarding its utility in AD remain limited [5, 6]. Similarly, optical coherence tomography (OCT) allows high-resolution quantitative analysis of retinal layer thicknesses, including macular and peripapillary retinal nerve fiber layers (RNFL) [7]. While some studies report significant inner retinal thinning in AD, these structural findings vary considerably among different cohorts [8].

This study aimed to investigate functional and structural retinal changes in AD by evaluating ERG parameters, together with OCT-based retinal layer thickness measurements. We aimed to identify retinal biomarkers that could provide noninvasive insights into retinal neurodegeneration associated with AD.

## Methods

All procedures involving human participants were conducted in accordance with the ethical standards of the Erciyes University Local Ethics Committee and the 1964 Helsinki Declaration and its later amendments or comparable ethical standards. The study protocol was approved by the Erciyes University Local Ethics Committee (Approval protocol No: 2021/566). Written informed consent was obtained from all individual participants included in the study.

### Details of the participants

This retrospective, cross-sectional study included 30 patients diagnosed with AD (59 eyes) and 31 age- and sex-matched healthy controls (31 eyes). Both eyes of patients with Alzheimer’s disease (AD) were included to preserve all available electrophysiological data, given the known inter-eye variability and potential asymmetry of retinal involvement in neurodegenerative diseases. In contrast, only one eye per control participant was analyzed to maintain independence of observations and avoid inflation of the effective sample size; when recordings from both eyes met the inclusion criteria, one eye was randomly selected using a random-number table, with odd digits indicating selection of the right eye and even digits indicating selection of the left eye. If the randomly selected eye did not meet predefined recording-quality criteria, the fellow eye was included in the analysis.

Control group exclusion criteria were diabetes mellitus, cerebrovascular disease, demyelinating disorders, significant head trauma, psychiatric illness, malignancy, use of sedative or neuroactive medications, and poor cooperation during testing. Ocular exclusion criteria were best-corrected visual acuity (BCVA) below 0.3 decimal (Snellen), dense media opacity, ocular trauma or recent surgery (e.g., cataract, vitreoretinal), optic nerve pathology, and refractive errors over ± 6 diopters.

AD diagnosis was confirmed by a neurologist at the Memory Disorders Outpatient Unit using the National Institute on Aging-Alzheimer’s Association (NIA-AA) criteria [9]. Cognitive status was assessed via the Mini-Mental State Examination (MMSE), scored from 0 (most severe) to 30 (normal), categorizing AD severity as mild (21–26), moderate (10–20), or severe (< 10) [10]. All AD patients were under pharmacological treatment. Most patients were using a cholinesterase inhibitor (donepezil or rivastigmine) together with memantine, and several patients were additionally receiving quetiapine for behavioural symptoms. Exclusion criteria for AD patients included diabetes mellitus, stroke, substance abuse, major psychiatric disorder, traumatic brain injury, other neurodegenerative disorders, and visual or motor impairments affecting testing reliability. Major psychiatric disorder was defined as any Diagnostic and Statistical Manual of Mental Illnesses (DSM-5) diagnosis associated with significant functional or cognitive impairment, including major depressive disorder, bipolar disorder, psychotic disorders, or schizophrenia.

### Examination and OCT-scanning

All participants underwent ophthalmic evaluation including best-corrected visual acuity (BCVA), slit-lamp biomicroscopy, intraocular pressure (IOP) measurement, autorefraction, and dilated fundus examination. Spectral-domain optical coherence tomography (OCT; Spectralis OCT, Heidelberg Engineering, Germany) was used to measure thicknesses of retinal layers, including nerve fiber layer (RNFL), ganglion cell layer (GCL), inner plexiform layer (IPL), inner nuclear layer (INL), outer plexiform layer (OPL), outer nuclear layer (ONL), and retinal pigment epithelium (RPE). Thicknesses were assessed within ETDRS subfields (central 1 mm, and superior, inferior, nasal, temporal sectors of the 1–3 mm ring). Sectoral peripapillary RNFL thicknesses were also recorded.

### Electroretinography assessment

Full-field ERG and photopic negative response (PhNR) recordings were obtained using the Metrovision Monpack 3 system (Metrovision, France) in accordance with the International Society for Clinical Electrophysiology of Vision (ISCEV) extended PhNR protocol [11]. Pupillary dilation was achieved with 1% tropicamide and 2.5% phenylephrine. Following 10 min of light adaptation, topical anesthesia (0.5% proparacaine) was administered, and single-use ERG-Jet contact lens electrodes were placed.

**Fig. 1:**
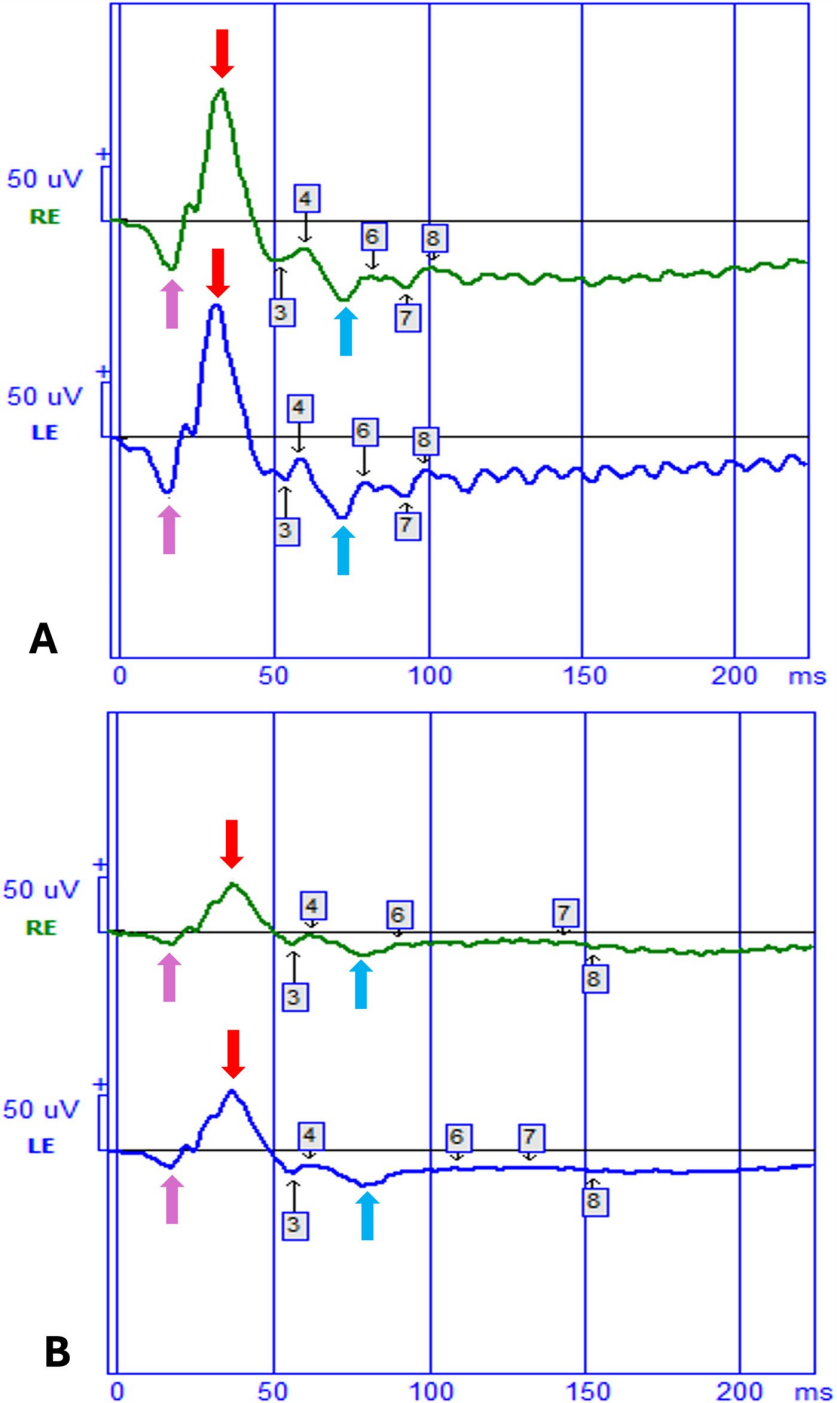
Photopic full-field electroretinogram (ERG) waveforms recorded from the right eye (RE, green traces) and left eye (LE, blue traces) using the red on blue PhNR extended protocol are shown. The lower panel **B** shows responses from a patient with Alzheimer’s disease, while the upper panel **A** shows responses from a healthy control. The a-wave (purple arrows) reflects the initial negative deflection mainly generated by photoreceptors. The b-wave (red arrows) is the subsequent positive peak arising from bipolar and Müller cell activity. The PhNR (Photopic Negative Response; blue arrows) is the slow negative component following the b-wave, originating primarily from retinal ganglion cells

All recordings were performed monocularly and sequentially, with the fellow eye occluded during each measurement to prevent contamination from consensual ERGs, in line with ISCEV protocols. Stimuli consisted of short-duration (< 5 ms) red flashes (630 nm; 1.7 cd·s/m^2^) presented on a steady blue background (465 nm; 8 cd/m^2^), with an inter-stimulus interval of 500 ms. Signals were amplified (× 1000), band-pass filtered (0.3–300 Hz), and averaged over 100 sweeps. Amplitudes and peak times (implicit times or latency) of the a-wave, b-wave, and PhNR were recorded. PhNR amplitude was quantified from baseline to the negative trough following the b-wave, typically occurring between 65 and 75 ms post-stimulus (Fig. 1), in accordance with ISCEV extended protocol recommendations.

Post-dilation pupil diameter was comparable between groups, with a mean diameter of approximately 7–8 mm. Palpebral aperture was continuously monitored throughout each recording session, and any trials showing partial eyelid closure or shadowing were excluded from analysis. ERG-Jet electrodes were carefully centered on the cornea under direct visualization and maintained with a sterile viscoelastic lubricant to ensure tear film uniformity. Recordings were paused for electrode rehydration or repositioning if centration shifted, and no electrode dislocation events occurred during the study.

Electrode impedance was maintained below 5 kΩ in all recordings. Traces contaminated by blink or motion artifacts were excluded. Eyes with significant media opacity or poor fixation were not included. Illumination and stimulus conditions were strictly standardized across all participants to minimize technical variability. The baseline-to-trough method was consistently used for PhNR amplitude measurement to reduce inter-method variability.

### Statistical analysis

Statistical analysis was performed using Jamovi (version 2.3.28). Normality of continuous variables was assessed using the Shapiro–Wilk test. Group comparisons between AD patients and controls were conducted using the independent samples t-test for normally distributed variables and the Mann–Whitney U test for non-normally distributed variables. To control for multiple comparisons, p-values were adjusted using the Bonferroni correction, with statistical significance defined accordingly. Categorical variables were compared using the chi-square test. Because both eyes were included for AD participants while only one eye per control participant was analyzed, generalized estimating equations (GEE) were used to account for within-subject interocular correlation in all inferential analyses. Participant identifier was specified as the clustering variable, allowing control participants to contribute a single independent observation and AD participants to contribute two correlated observations within the same analytical framework. Receiver operating characteristic (ROC) curve analyses were also conducted using GEE-based models to appropriately account for inter-eye correlation while preserving eye-level information. This approach avoided artificial inflation of diagnostic accuracy and was preferred over per-participant averaging, as it retains biologically relevant interocular variability. Additionally, inter-eye comparisons (oculus dexter [OD] vs oculus sinister [OS]) were assessed using a linear mixed-effects model. The area under the curve (AUC) is reported with 95% confidence intervals. A *p*-value < 0.05 was considered statistically significant.

## Results

**Fig. 2:**
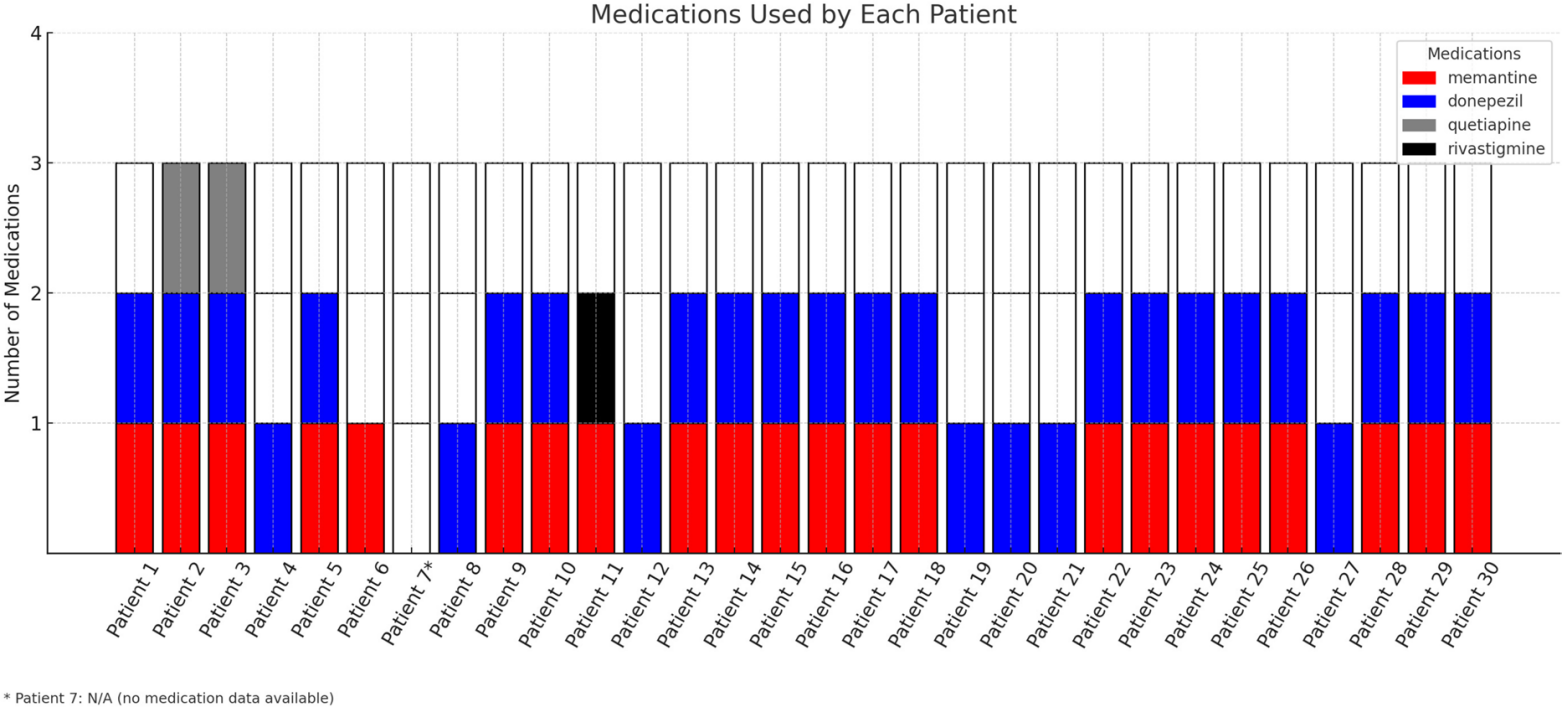
The chart shows the drugs used by each patient. Each color represents a specific medication: red for memantine, blue for donepezil, gray for quetiapine, and black for rivastigmine

A total of 90 eyes (59 eyes from 30 AD patients, 31 eyes from 31 controls) were included. There were no significant differences between AD and control groups regarding gender (F/M; AD: 16/14, control: 13/18, *p* = 0.339), age (AD: 71.7 ± 8.9 years, control: 69.4 ± 8.5 years, *p* = 0.287), IOP (AD: 14.6 ± 3.1 mmHg, control: 14.6 ± 2.2 mmHg, *p* = 0.615), and BCVA (logMAR; AD: 0.84 ± 0.1, control: 0.96 ± 0.08, *p* = 0.269). Mean MMSE score in the AD group was 15.5 ± 3.9, and mean disease duration was 2.67 ± 1.5 years (Table 1). A detailed, patient-by-patient distribution of medications is presented in Fig. 2.

**Table 1:** Electrophysiological and structural retinal findings in Alzheimer’s disease: demographic and clinical characteristics of study participants: demographics and basic clinical parameters

Variables	Control (n = 31)	Alzheimer’s Disease (n = 30)	p
Gender (F/M)	13/18	16/14	0.339
Age (years)	69.4 ± 8.5	71.7 ± 8.9	0.287
IOP (mmHg)	14.6 ± 2.2	14.6 ± 3.1	0.615
BCVA (logMAR)	0.96 ± 0.08	0.84 ± 0.1	0.269
Alzheimer's disease MMSE score	–	15.5 ± 3.9	–
Alzheimer's disease Duration (years)	–	2.67 ± 1.5	–

MMSE: Mini‐Mental State Examination; IOP: intraocular pressure; BCVA: best corrected visual acuity

Because age is known to influence ERG amplitudes physiologically [12], age was included as a covariate in all GEE analyses. In age adjusted analysis, ERG results revealed significantly lower amplitudes in AD patients compared to controls: a-wave (AD: − 13.4 ± 11.9 µV, control: − 32.5 ± 13.7 µV, *p* < 0.001), b-wave (AD: 59.1 ± 28 µV, control: 156 ± 48.8 µV, *p* < 0.001), and PhNR amplitude (AD: − 21.4 ± 14.9 µV, control: − 51 ± 15.4 µV, *p* < 0.001). There were no significant differences in peak times (implicit times) for the a-wave (AD: 15.6 ± 5.35 ms, control: 16.0 ± 1.37 ms, *p* = 0.218), b-wave (AD: 34.1 ± 3.58 ms, control: 32.5 ± 1.45 ms, *p* = 0.502), or PhNR (AD: 74 ± 5.84 ms, control: 71.9 ± 3.27 ms, *p* = 0.438). Amplitude ratios (PhNR/b-wave) were also similar between groups (AD: − 0.545 ± 0.42, control: − 0.432 ± 0.13, *p* = 0.75) (Table 2). In unadjusted analyses, b-wave implicit time was significantly prolonged in the AD group compared with controls (*p* = 0.03). No significant unadjusted between-group differences were observed for other ERG parameters. However, after adjustment for age, the between-group difference in b-wave implicit time was attenuated and no longer statistically significant, indicating that age-related variability may partially account for the observed difference in implicit time. In addition, the distributions of a-wave, b-wave, and PhNR amplitudes and implicit times, as well as their inter-relationships, are illustrated in Supplementary Fig. 1. In an additional inter-eye comparison analysis (OD vs OS), significant differences were observed for a-wave peak time, b-wave amplitude, IPL nasal thickness, and INL inferior thickness (Supplementary file 2).

**Table 2:** Electrophysiological and structural retinal findings in Alzheimer’s disease: ERG Amplitudes and Implicit Times

Parameter	Alzheimer’s disease (n = 59 eyes)	Control (n = 31eyes)	*p*
a wave amplitude (µV)	− 13.4 ± 11.9	− 32.5 ± 13.0	< 0.001*
a wave implicit time (ms)	15.6 ± 5.3	16.0 ± 1.3	0.284
b wave amplitude (µV)	59.1 ± 28.0	156 ± 48.8	< 0.001*
b wave implicit time (ms)	34.1 ± 3.58	32.5 ± 1.4	0.473
PhNR wave amplitude (µV)	− 21.4 ± 14.9	− 51.0 ± 15.4	< 0.001*
PhNR wave implicit time (ms)	74.0 ± 5.8	71.9 ± 3.2	0.438
Amplitude ratio (PhNR/b wave)	− 0.54 ± 0.4	− 0.43 ± 0.1	0.750

ms: milliseconds, µV: microvolt, PhNR: photopic negative response, ERG: electroretinogram

* Statistically significant

OCT analysis revealed selective differences in retinal layer thicknesses between AD and control groups. Significant thinning was observed in the central inner nuclear layer (INL; AD: 25 ± 6.6 µm, control: 25.2 ± 8.2 µm, *p* = 0.041), inferior outer plexiform layer (OPL; AD: 32.7 ± 6.2 µm, control: 36.9 ± 8.9 µm, *p* = 0.01), and temporal OPL (AD: 29.3 ± 5.2 µm, control: 34.8 ± 7 µm, *p* = 0.001). Other retinal layers, including RNFL, GCL, IPL, RPE, and peripapillary RNFL thicknesses (pRNFLT), showed no significant differences between groups (Table 3).

**Table 3:** Electrophysiological and structural retinal findings in Alzheimer’s disease: OCT parameters

Variables		Control (n = 31)	Alzheimer’s Disease (n = 59)	*p*
NFL (μm)	Superior	26.0 ± 5.7	24.8 ± 5.0	0.106
	Inferior	27.1 ± 4.0	28.5 ± 7.3	0.679
	Central	13.7 ± 3.0	13.6 ± 3.0	0.837
	Temporal	19.3 ± 2.1	20.0 ± 4.4	0.985
	Nasal	23.1 ± 3.8	23.3 ± 5.2	0.341
GCL (μm)	Superior	52.7 ± 6.1	47.5 ± 8.1	0.203
	Inferior	51.5 ± 5.7	48.4 ± 6.9	0.832
	Central	18.3 ± 6.4	16.9 ± 5.7	0.215
	Temporal	46.2 ± 5.8	43.1 ± 7.2	0.848
	Nasal	51.3 ± 6.6	47.4 ± 6.7	0.269
IPL (μm)	Superior	41.6 ± 5.1	37.8 ± 5.6	0.318
	Inferior	40.3 ± 4.0	38.1 ± 5.1	0.964
	Central	22.5 ± 5.0	22.1 ± 5.0	0.560
	Temporal	41.3 ± 3.2	38.7 ± 5.5	0.296
	Nasal	42.6 ± 4.9	39.5 ± 5.3	0.272
INL (μm)	Superior	42.0 ± 4.6	40.0 ± 5.9	0.353
	Inferior	41.9 ± 4.7	39.0 ± 4.5	0.260
	Central	25.2 ± 8.2	25.0 ± 6.6	0.041*
	Temporal	38.8 ± 5.0	37.2 ± 5.1	0.708
	Nasal	41.7 ± 3.8	40.2 ± 4.8	0.386
OPL (μm)	Superior	36.0 ± 9.1	35.2 ± 6.5	0.856
	Inferior	36.9 ± 8.9	32.7 ± 6.2	0.011*
	Central	29.8 ± 8.1	27.7 ± 6.2	0.105
	Temporal	34.8 ± 7.0	29.3 ± 5.2	0.001*
	Nasal	38.0 ± 7.4	35.5 ± 8.0	0.410
ONL (μm)	Superior	62.6 ± 12.7	62.6 ± 10.5	0.265
	Inferior	61.9 ± 11.7	60.7 ± 12.8	0.411
	Central	80.6 ± 15.0	80.3 ± 15.6	0.240
	Temporal	66.0 ± 11.8	68.0 ± 10.5	0.053
	Nasal	65.8 ± 12.2	64.7 ± 12.3	0.419
RPE (μm)	Superior	14.9 ± 2.0	14.9 ± 1.8	0.747
	Inferior	14.1 ± 1.5	14.5 ± 2.6	0.536
	Central	15.8 ± 14.9	16.8 ± 14.9	0.130
	Temporal	14.3 ± 1.9	15.7 ± 7.0	0.301
	Nasal	14.8 ± 1.8	15.4 ± 2.9	0.174
pRNFLT (μm)	Temporal-Superior	134.0 ± 19.6	136.0 ± 32.4	0.183
	Nasal-Superior	117.0 ± 23.2	110.0 ± 24.7	0.815
	Temporal	69.3 ± 8.1	74.3 ± 12.6	0.137
	Nasal	75.2 ± 14.3	75.4 ± 16.9	0.199
	Temporal-Inferior	143.0 ± 18.4	138.0 ± 22.5	0.707
	Nasal-Inferior	107.0 ± 19.1	114.0 ± 32.6	0.120

OPL: outer plexiform layer; INL: inner plexiform layer; NFL: nerve fiber layer; GCL: ganglion cell layer; IPL: inner plexiform layer; ONL: outer nuclear layer; RPE: retinal pigment epithelium layer; pRNFLT: peripapillary retinal nerve fiber layer thickness, μm: micron

^*^ statistically significant

**Fig. 3:**
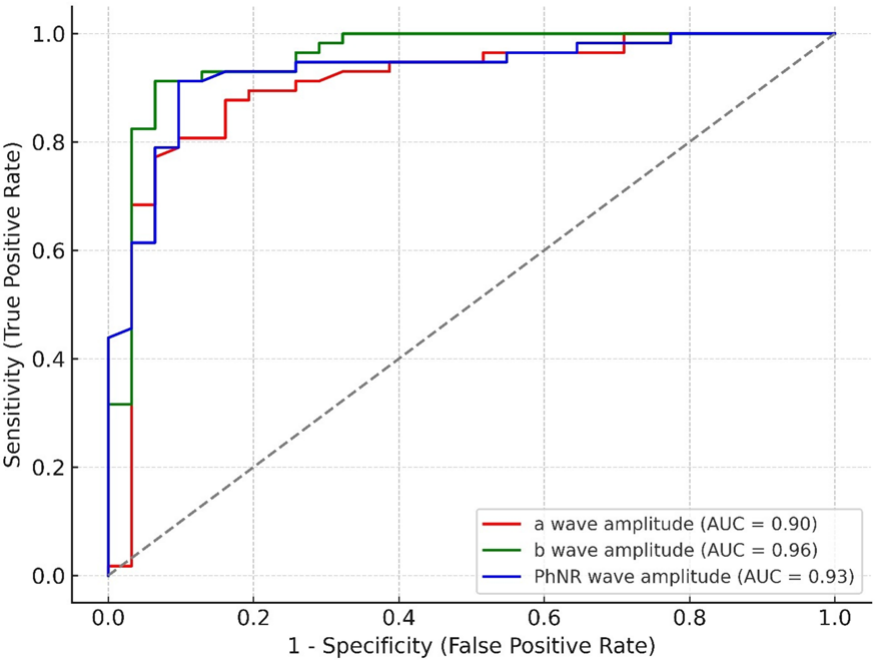
Receiver operating characteristic (ROC) curves illustrating the diagnostic performance of a-wave amplitude (red), b-wave amplitude (green), and photopic negative response (PhNR) amplitude (blue) in differentiating patients with Alzheimer’s disease from healthy controls. The y-axis represents sensitivity (%), while the x-axis represents 1 − specificity (%)

In ROC analysis, the b-wave amplitude demonstrated the highest diagnostic performance among ERG parameters (AUC = 0.956), followed by PhNR amplitude (AUC = 0.930). a-wave amplitude also demonstrated strong discrimination (AUC: 0.904, sensitivity: 87.7%, specificity: 83.9%). In addition, b-wave amplitude had high classification power (AUC: 0.956, sensitivity:91.2%, specificity:93.5%) (Table 4). ROC curves are presented in Fig. 3.

**Table 4:** Electrophysiological and structural retinal findings in Alzheimer’s disease: receiver operating characteristic (ROC) analysis of ERG parameters

Variables	Sensitivity (%)	Specificity (%)	Area under curve	Youden's index	*p*
a-wave amplitude	87.7	83.9	0.904	0.716	0.007*
b-wave amplitude	91.2	93.5	0.956	0.848	< 0.001*
PhNR amplitude	91.2	90.3	0.930	0.816	< 0.001*

PhNR: photopic negative response

* Statistically significant

## Discussion

This study demonstrated significant functional and structural retinal alterations in patients with AD. PhNR, a-wave, and b-wave amplitudes were markedly reduced in the AD group, indicating that retinal dysfunction in AD is not limited to ganglion cells but involves multiple retinal layers, including photoreceptors and bipolar cells. These electrophysiological changes occurred despite preserved visual acuity, suggesting subclinical and widespread retinal involvement.

Our ROC analysis demonstrated high diagnostic accuracy for all three ERG parameters evaluated. Notably, the b-wave amplitude showed the highest discriminative power with an AUC of 0.95, followed by the PhNR amplitude (AUC = 0.93) and the a-wave amplitude (AUC = 0.90). These findings suggest that inner retinal dysfunction, particularly at the level of bipolar and ganglion cells, may be reliably detected in AD patients using full-field ERG. The strong performance of the PhNR amplitude further reinforces its value as a potential functional biomarker of retinal ganglion cell impairment in AD. Given the non-invasive nature and high reproducibility of ERG testing, these results may support its potential role in the early detection or monitoring of retinal involvement in neurodegenerative conditions such as AD.

Neurodegenerative diseases such as AD present complex and still poorly understood pathophysiological mechanisms. Several studies have demonstrated widespread electrophysiological alterations in AD, involving various brain regions. Yu et al. [13] reported frequent subclinical epileptiform discharges in the temporal cortex, supporting the concept of cortical hyperexcitability. Cheron et al. [14] identified functional impairments in cerebellar Purkinje neurons, while Babiloni et al. [15] showed that EEG slowing, particularly in the alpha band, is an early biomarker of cognitive decline in AD. Given the electrophysiological nature of these central findings, it is important to explore whether similar dysfunctions can also be detected at the level of the visual system, particularly in the retina and optic nerve. Previous ERG and VEP studies have shown that various alterations can occur in patients with AD. Asanad et al. [7] also reported a significant reduction in PhNR amplitude (50.1% reduction, *p* = 0.003) in cognitively healthy individuals with pathological cerebrospinal fluid Aβ42/Tau ratios (CH-PATs) compared to those with normal ratios (CH-NATs), despite no significant differences in PhNR peak times between the groups. This finding is in line with our results, which showed decreased PhNR amplitudes in AD patients without significant implicit times. In contrast to our findings, Katz et al. [16] did not observe significant differences in flash ERG parameters (a-wave and b-wave amplitudes and latencies) between AD patients and healthy controls. This discrepancy may be attributed to the small sample size in their study (n = 6 per group), which likely limited the statistical power to detect subtle electrophysiological changes. The electrophysiological changes in AD may reflect underlying neurodegenerative mechanisms associated with AD pathology, such as beta-amyloid accumulation and cholinergic deficits, which have been observed not only in the brain but also in the retina and optic nerve [17]. In addition, the widespread retinal neurodegeneration may align with previous findings of retinal beta-amyloid deposition and cholinergic dysfunction, both of which can affect outer and inner retinal layers [18]. Notably, the reduction in PhNR amplitude highlights early retinal ganglion cell impairment and may serve as a potential functional biomarker of AD-related neurodegeneration.

Our findings of reduced PhNR amplitudes in AD are consistent with a broader body of evidence demonstrating that neurodegenerative and neuropsychiatric disorders affecting central neural pathways also manifest selective retinal dysfunction. The seminal work of Viswanathan et al. [19] established the PhNR as a highly sensitive indicator of retinal ganglion cell activity, with marked reduction even in early glaucoma. Similarly, recent studies have reported PhNR attenuation in schizophrenia accompanied by structural GCL thinning, suggesting parallel functional and anatomical compromise [20, 21]. Parkinson’s disease cohorts likewise exhibit impaired PhNR and scotopic b-wave amplitudes, detectable even at early disease stages, supporting the retina’s role as an early biomarker of CNS degeneration [22]. In contrast, the preservation of PhNR in autism spectrum disorder indicates that retinal ganglion cell dysfunction is not universal across neurological conditions, underscoring the specificity of PhNR reduction in disorders characterized by ganglion cell vulnerability such as AD [6]. Taken together with prior evidence from other neurodegenerative and neuropsychiatric disorders, our findings indicate that PhNR reduction in AD may reflect a shared pathway of inner retinal vulnerability involving retinal ganglion cells. Moreover, retinal function may provide an accessible and sensitive indicator of AD-related neurodegeneration.

Given the anatomical and developmental parallels between the retina and the brain, increasing attention has been directed toward the retina as a potential window into cerebral neurodegeneration [23, 24]. Hallmark AD pathologies, including amyloid-β plaques and hyperphosphorylated tau, have been detected not only in the brain but also in the retinal ganglion cell layer (GCL) and retinal nerve fiber layer (RNFL) [25]. Furthermore, mechanisms such as microvascular dysfunction, oxidative stress, and neuroinflammation—commonly implicated in AD—are also thought to contribute to retinal changes [25–27]. In our study, significant thinning was observed particularly in the INL and OPL layers of AD patients. These findings are in line with the meta-analysis by Chan et al. [28], which demonstrated reductions in GC-IPL, GCC, and RNFL thickness in AD. However, our study differs by focusing on less commonly evaluated inner retinal layers, such as the INL and OPL. In addition, our findings did not reveal significant alterations in most other retinal parameters, suggesting that detectable retinal involvement may not be present in all cases of AD.

In the study by Lim et al. [29], the 5xFAD mouse model—which recapitulates key pathological features of Alzheimer’s disease—also demonstrates reductions in a- and b-wave amplitudes at later disease stages, consistent with the ERG depression observed in our AD cohort. Structurally, 5xFAD mice show inner retinal involvement, including RNFL thinning and synaptic-layer alterations, particularly within the inner plexiform layer during early stages and extending outward as the disease progresses. In our patients, no widespread structural thinning was detected; however, significant reductions were observed in the OPL and central INL, suggesting selective mid-retinal vulnerability. Such discrepancies may indicate that retinal dysfunction evolves differently in human AD than in transgenic models, potentially reflecting the longer disease duration, and greater biological complexity seen in patients.

Cholinesterase inhibitors (e.g., donepezil, rivastigmine) and NMDA receptor antagonists (e.g., memantine) have been shown to modulate cortical electrophysiological activity in Alzheimer’s disease, with EEG studies reporting reductions in pathological theta activity and improvements in cognitive performance [30–32]. However, their effects on retinal function remain less well defined. Although the presence of cholinergic and glutamatergic pathways in the inner retina suggests potential susceptibility to these agents [33, 34], direct evidence for retinal neuroprotective effects is limited. In our study, ERG responses—including PhNR, a-wave, and b-wave amplitudes—were significantly reduced in patients receiving these medications. Importantly, the independent contributions of disease-related retinal neurodegeneration and medication-related effects cannot be disentangled in this cross-sectional design. In particular, cholinesterase inhibitors such as donepezil have been reported to influence ocular physiology, including pupil dynamics and intraocular pressure, which may in turn affect ERG amplitudes [35]. Therefore, the observed ERG reductions may reflect a combination of underlying retinal pathology and medication-mediated effects, rather than disease severity alone. Accordingly, any interpretation regarding potential neuroprotective or modulatory effects of AD medications on retinal electrophysiology should be made with caution. Longitudinal studies and medication-stratified analyses will be required to clarify the retinal effects of these agents and to distinguish treatment-related influences from disease-related changes.

This study has several limitations. The relatively small sample size may limit generalizability. The cross-sectional design precludes evaluation of longitudinal changes. Additionally, only one eye per control participant was included, whereas both eligible eyes were analyzed in the AD group; although GEE modelling accounted for inter-eye correlation, this may have reduced symmetry in eye-level sampling between groups. Notably, supplementary inter-eye analyses within the AD group demonstrated statistically significant OD–OS differences in selected electrophysiological and OCT parameters, supporting measurable interocular variability in this cohort. Moreover, the lack of cerebrospinal fluid or neuroimaging data prevents correlation between retinal findings and established biomarkers of AD. Furthermore, all participants in the AD group were under pharmacological treatment, which could potentially influence electrophysiological and structural retinal measurements; thus, medication effects cannot be entirely excluded. Future studies should consider larger, medication-naïve populations and longitudinal designs to better delineate the specific retinal changes attributable to AD itself, independent of pharmacological intervention. Finally, the a- and b-wave measurements in this study were obtained using a red-on-blue PhNR protocol rather than standard white-flash photopic ERG. Long-wavelength stimuli are known to depress photopic ON-pathway responses and modify b-wave morphology, which may contribute to the overall reduction of a- and b-wave amplitudes observed in our cohort [36]. Therefore, our a- and b-wave findings should be interpreted within the context of PhNR-optimized chromatic stimulation and cannot be directly compared with conventional white-flash recordings. Future studies incorporating light-adapted 3.0 cd·s/m^2^ white-flash ERG protocols may allow a more comprehensive assessment of global photoreceptor and bipolar cell function. Another limitation is the lack of pharmacological stratification. Most AD participants were receiving cholinesterase inhibitors, and medications such as donepezil are known to cause mild miosis and reduce retinal illuminance, potentially leading to lower ERG amplitudes [35]. Thus, part of the observed ERG attenuation in the AD group may reflect medication effects rather than disease-related retinal dysfunction alone. Future studies should include medication-naïve patients or stratified analyses to disentangle pharmacological and neurodegenerative contributions. Moreover, although pharmacologic dilation and standardized electrode placement procedures were used, we cannot entirely exclude subtle between-group differences in pupil diameter, eyelid aperture, tear film quality, or Jet-electrode centration. Such factors may contribute to the globally reduced a-, b- and PhNR amplitudes observed in the AD group, and future studies incorporating continuous pupil tracking and objective electrode-position monitoring are warranted.

In conclusion, our findings demonstrate significantly reduced amplitudes of PhNR, a-wave, and b-wave in patients with AD, indicating that retinal dysfunction extends beyond ganglion cells to involve multiple retinal layers, including photoreceptors and bipolar cells. The high diagnostic accuracy of PhNR amplitude highlights its potential as an early functional biomarker for detecting retinal ganglion cell impairment associated with AD. Structurally, selective thinning in the INL and OPL was observed, further suggesting specific retinal layer involvement. Therefore, combined ERG and OCT evaluation appears promising as a non-invasive approach for detecting retinal neurodegeneration in AD patients.

## Supplementary Information

Below is the link to the electronic supplementary material.

Supplementary file1: Supplementary Figure 1: (A) Group-wise distributions of ERG peak times (a-wave, b-wave, and PhNR) in Alzheimer’s disease (AD) and control groups. (B) Corresponding distributions of a-wave, b-wave, and PhNR amplitudes across groups. Box plots show median and interquartile range, with individual data points overlaid. (C) Scatter plots illustrating the inter-relationships between a-wave amplitude and PhNR amplitude (left), and between b-wave amplitude and PhNR amplitude (right), with colors indicating group membership (control vs. AD)

Supplementary file2 (DOCX 37 KB)

## Data Availability

The datasets generated during and/or analyzed during the current study are available from the corresponding author on reasonable request.

## Declarations

### Statement on the welfare of animals

No animals were used in this research.
