# Supplementary figures and images for: Functional and structural retinal alterations in Alzheimer’s disease: insights from photopic negative response (PhNR) and OCT-based analysis
Source: Doc Ophthalmol. 2026 Mar 4;152(3):369–80. doi: 10.1007/s10633-026-10089-8 (PMC13194298; doi:10.1007/s10633-026-10089-8)

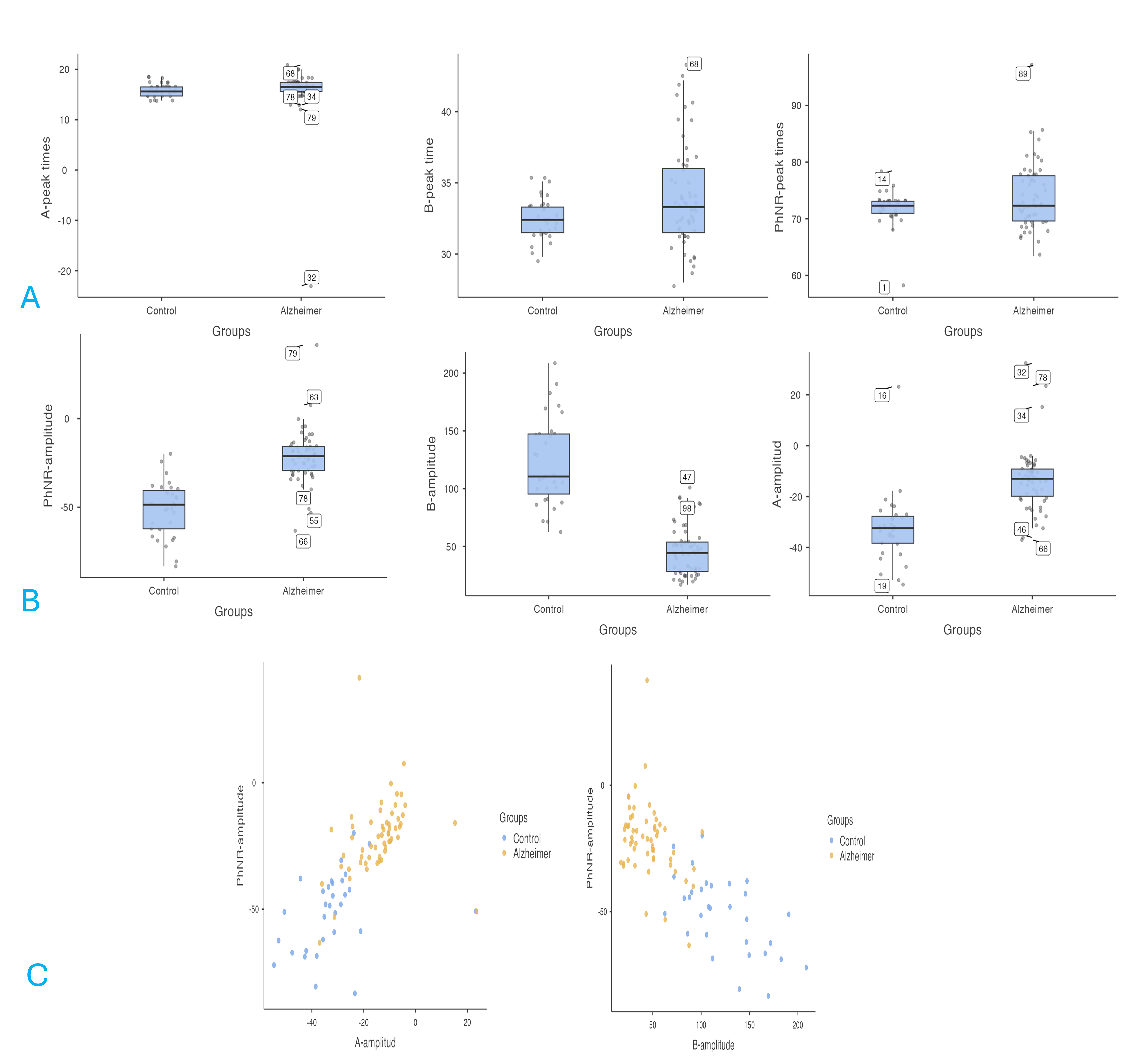

Supplement: Supplementary file 1 — Supplementary file1: Supplementary Figure 1: (A) Group-wise distributions of ERG peak times (a-wave, b-wave, and PhNR) in Alzheimer’s disease (AD) and control groups. (B) Corresponding distributions of a-wave, b-wave, and PhNR amplitudes across groups. Box plots show median and interquartile range, with individual data points overlaid. (C) Scatter plots illustrating the inter-relationships between a-wave amplitude and PhNR amplitude (left), and between b-wave amplitude and PhNR amplitude (right), with colors indicating group membership (control vs. AD) [file 10633_2026_10089_MOESM1_ESM.png]
