# Supplementary material for: Functional and structural retinal alterations in Alzheimer’s disease: insights from photopic negative response (PhNR) and OCT-based analysis
Source: Doc Ophthalmol. 2026 Mar 4;152(3):369–80. doi: 10.1007/s10633-026-10089-8 (PMC13194298; doi:10.1007/s10633-026-10089-8)
Supplement: Supplementary file 2 — Supplementary file2 (DOCX 37 KB) [file 10633_2026_10089_MOESM2_ESM.docx]

| **Table 4**. Inter-eye comparison of electrophysiological and OCT-derived retinal layer measurements | | | | | | | | | |
| --- | --- | --- | --- | --- | --- | --- | --- | --- | --- |
| **Parameters** | | **Estimate (OS−OD)** | **SE** | **95% CI (lower to upper)** | **t** | | ***p*** | | |
| **a-wave** | *peak time (ms)* | -0.749 | 0.186 | -1.13 to -0.37 | -4.03 | | <0.001* | | |
|  | *amplitude(µV)* | -2.80 | 2.54 | -7.94 to 2.34 | -1.10 | | 0.286 | | |
| **b-wave** | *peak time (ms)* | -0.521 | 0.386 | -1.30 to 0.26 | -1.35 | | 0.193 | | |
|  | *amplitude(µV)* | 6.97 | 2.18 | 2.55 to 11.4 | 3.19 | | 0.005* | | |
| **PhNR** | *peak time (ms)* | 0.493 | 0.840 | -1.21 to 2.20 | 0.59 | | 0.564 | | |
|  | *amplitude(µV)* | 1.39 | 5.35 | -9.46 to 12.2 | 0.26 | | 0.797 | | |
| **PhNR ratio** | *PhNR / b wave* | 0.072 | 0.150 | -0.23 to 0.38 | 0.48 | | 0.635 | | |
| **NFL(μm)** | *Central* | -0.380 | 0.521 | -1.45 to 0.69 | -0.729 | | 0.478 | | |
|  | *Superior* | -2.31 | 1.66 | -5.71 to 1.09 | -1.39 | | 0.184 | | |
|  | *Inferior* | -0.396 | 2.00 | -4.49 to 3.70 | -0.199 | | 0.845 | | |
|  | *Temporal* | -1.44 | 1.56 | -4.65 to 1.76 | -0.926 | | 0.370 | | |
|  | *Nasal* | 0.0003 | 1.25 | -2.56 to 2.56 | 0.00024 | | 1.000 | | |
| **GCL (μm)** | *Central* | -1.41 | 1.73 | -4.96 to 2.14 | -0.816 | | 0.428 | | |
|  | *Superior* | -0.667 | 1.70 | -4.15 to 2.82 | -0.392 | | 0.700 | | |
|  | *Inferior* | -0.288 | 1.05 | -2.43 to 1.86 | -0.275 | | 0.787 | | |
|  | *Temporal* | 0.840 | 1.31 | -1.86 to 3.54 | 0.639 | | 0.533 | | |
|  | *Nasal* | 1.78 | 1.54 | -1.37 to 4.93 | 1.16 | | 0.266 | | |
| **IPL (μm)** | *Central* | 1.78 | 1.46 | -1.22 to 4.79 | 1.22 | | 0.243 | | |
|  | *Superior* | 0.812 | 1.09 | -1.42 to 3.04 | 0.747 | | 0.467 | | |
|  | *Inferior* | -0.508 | 1.24 | -3.05 to 2.03 | -0.410 | | 0.688 | | |
|  | *Temporal* | -0.467 | 1.01 | -2.54 to 1.60 | -0.463 | | 0.650 | | |
|  | *Nasal* | 2.83 | 1.18 | 0.404 to 5.26 | 2.39 | | 0.031* | | |
| **INL (μm)** | *Central* | -1.82 | 1.40 | -4.70 to 1.06 | -1.29 | | 0.217 | | |
|  | *Superior* | 1.63 | 1.38 | -1.21 to 4.47 | 1.18 | | 0.257 | | |
|  | *Inferior* | 2.25 | 0.640 | 0.935 to 3.56 | 3.51 | | 0.003* | | |
|  | *Temporal* | 2.15 | 1.38 | -0.672 to 4.97 | 1.56 | | 0.139 | | |
|  | *Nasal* | 1.53 | 1.53 | -1.62 to 4.67 | 0.997 | | 0.336 | | |
| **OPL (μm)** | *Central* | 1.53 | 1.53 | -1.62 to 4.67 | 0.997 | | 0.336 | | |
|  | *Superior* | -1.57 | 1.83 | -5.33 to 2.19 | -0.85 | | 0.406 | |  |
|  | *Inferior* | 0.08 | 1.50 | -2.98 to 3.15 | 0.06 | | 0.957 | |  |
|  | *Temporal* | 1.40 | 1.16 | -0.98 to 3.77 | 1.21 | | 0.246 | |  |
|  | *Nasal* | -1.09 | 2.63 | -6.49 to 4.32 | -0.41 | | 0.685 | |  |
| **ONL (μm)** | *Central* | 3.77 | 2.18 | -0.71 to 8.24 | 1.73 | | 0.106 | |  |
|  | *Superior* | 3.40 | 2.56 | -1.85 to 8.66 | 1.33 | | 0.205 | |  |
|  | *Inferior* | 2.03 | 2.44 | -2.97 to 7.03 | 0.83 | | 0.418 | |  |
|  | *Temporal* | 0.94 | 1.76 | -2.67 to 4.55 | 0.53 | | 0.602 | |  |
|  | *Nasal* | -1.19 | 3.79 | -8.96 to 6.58 | -0.31 | | 0.758 | |  |
| **RPE (μm)** | *Central* | -2.66 | 2.02 | -6.81 to 1.50 | -1.31 | 0.209 | |  |  |
|  | *Superior* | -0.279 | 0.665 | -1.64 to 1.08 | -0.419 | 0.681 | |  |  |
|  | *Inferior* | -1.65 | 0.953 | -3.6 to 0.309 | -1.73 | 0.104 | |  |  |
|  | *Temporal* | -2.56 | 2.39 | -7.47 to 2.35 | -1.07 | 0.302 | |  |  |
|  | *Nasal* | -0.88 | 1.218 | -3.38 to 1.62 | -0.723 | 0.481 | |  |  |
| **pRNFLT (μm)** | *Temporal* | -3.88 | 2.63 | -9.22 to 1.46 | -1.47 | 0.158 | |  |  |
|  | *Inferior* | -1.79 | 2.59 | -7.05 to 3.46 | -0.692 | 0.497 | |  |  |
|  | *Temporal-Superior* | -3.6 | 4.66 | -13.0 to 5.84 | -0.772 | 0.449 | |  |  |
|  | *Nasal-Superior* | 4.83 | 4.5 | -4.28 to 13.9 | 1.07 | 0.295 | |  |  |
|  | *Temporal-Inferior* | 1.13 | 4.98 | -8.97 to 11.2 | 0.227 | 0.823 | |  |  |
|  | *Nasal-Inferior* | 6.17 | 6.83 | -7.67 to 20 | 0.90 | 0.378 | |  |  |

CI, confidence interval; PhNR, photopic negative response, OD: oculus dexter, OS: oculus sinister, OPL: outer plexiform layer; INL: inner plexiform layer; NFL: nerve fiber layer; GCL: ganglion cell layer; IPL: inner plexiform layer; ONL: outer nuclear layer; RPE: retinal pigment epithelium layer; pRNFLT: peripapillary retinal nerve fiber layer thickness, μm: micron, ms: milliseconds, µV: microvolt, * statistically significant
